# Estimation of ancestry from cranial measurements based on MDCT data acquired in a Japanese and Western Australian population

**DOI:** 10.1007/s00414-024-03159-6

**Published:** 2024-01-22

**Authors:** Suguru Torimitsu, Akari Nakazawa, Ambika Flavel, Lauren Swift, Yohsuke Makino, Hirotaro Iwase, Daniel Franklin

**Affiliations:** 1 Centre for Forensic Anthropology, University of Western Australia, Crawley, WA 6009 Australia; 2 Department of Forensic Medicine, Graduate School of Medicine, The University of Tokyo, Tokyo, 113-0033 Japan; 3 Department of Obstetrics and Gynecology, Graduate School of Medicine, The University of Tokyo, Tokyo, 113-8655 Japan

**Keywords:** Forensic anthropology, Multidetector computed tomography, Skull, Japanese, Western Australian, Ancestry assessment

## Abstract

The estimation of ancestry is important not only towards establishing identity but also as a required precursor to facilitating the accurate estimation of other attributes such as sex, age at death, and stature. The present study aims to analyze morphological variation in the crania of Japanese and Western Australian individuals and test predictive models based on machine learning for their potential forensic application. The Japanese and Western Australian samples comprise computed tomography (CT) scans of 230 (111 female; 119 male) and 225 adult individuals (112 female; 113 male), respectively. A total of 18 measurements were calculated, and machine learning methods (random forest modeling, RFM; support vector machine, SVM) were used to classify ancestry. The two-way unisex model achieved an overall accuracy of 93.2% for RFM and 97.1% for SVM, respectively. The four-way sex and ancestry model demonstrated an overall classification accuracy of 84.0% for RFM and 93.0% for SVM. The sex-specific models were most accurate in the female samples (♀ 95.1% for RFM and 100% for SVM; ♂91.4% for RFM and 97.4% for SVM). Our findings suggest that cranial measurements acquired in CT images can be used to accurately classify Japanese and Western Australian individuals into their respective population. This is the first study to assess the feasibility of ancestry estimation using three-dimensional CT images of the skull.

**Supplementary Information:**

The online version contains supplementary material available at 10.1007/s00414-024-03159-6.

## Introduction

Establishing the identity of unidentified human remains is of fundamental importance in a forensic investigation, particularly in the analysis of dismembered, burned, or severely mutilated corpses or skeletal remains [1]. Although estimating ancestry is especially challenging [2], ancestry is an integral parameter not only to assist identification efforts directly but also as a required precursor to estimating sex, age at death, stature, and other attributes using population specific data [3].

It is generally accepted that the skull, especially the midface, is the most diagnostic region of the skeleton for estimating ancestry [4, 5]. There are two main methodological approaches typically applied in the anthropological assessment: morphoscopic (visual or non-metric) and morphometric. Procedures for estimating ancestry, whatever the statistical treatment, focus on non-metric or metric features, based on appreciable and/or significant cranial diversity between global populations [4]. Although non-metric approaches lack objectivity and require more experience, metric methods have less so, largely because individual cranial measurements are clearly defined on the basis of established craniometrics landmarks [3].

Ancestry estimation based on linear discriminant analysis (LDA) is one of the most commonly applied statistical approaches; computer applications, such as FORDISC [6, 7] and CRANID [8, 9], simplify the use of LDA for ancestry estimation, and the associated output includes statistical quantification of accuracy (e.g., posterior and typicality probabilities) that are useful for interpretation and decision-making. In addition, a machine learning modeling technique for ancestry estimation on the basis of skeletal metric data has been proposed [10, 11]. However, it has been reported that American Southwest Hispanic skulls are often misclassified as Asians, in particular Japanese, when performing ancestry estimation using craniometric data [12]. Thus, it is important that crania from other global populations are examined and compared to those originating from Japan, to minimize the possibility of misclassifications.

Computed tomography (CT) clearly depicts bone structures [13, 14]. In addition, it is known that bone measurements in CT images can be acquired with the same level of accuracy as those from real bone specimens [15, 16]. Importantly, the requisite data for calculating predictive models for estimating biological attributes associated with a routine anthropological assessment can be effectively developed using data acquired in CT images [15, 17, 18]. However, to date, no study has examined the feasibility of ancestry estimation using CT scanning techniques.

The aim of the present study, therefore, is to explore morphological variances between crania from contemporary Japanese and Western Australian populations and thereafter assess the feasibility of ancestry classification on the basis of morphometric data acquired in multidetector CT (MDCT) images using machine learning statistical approaches.

## Materials and methods

### Materials

#### Japanese population

The sample comprises postmortem CT (PMCT) scans of 230 adult corpses of known age and sex (111 female, mean age 48.96 ± 18.08 years; 119 male, mean age 46.80 ± 18.39 years) at the Department of Forensic Medicine at the University of Tokyo between July 2017 and May 2022. The estimated postmortem interval for all subjects was <14 days. The exclusion criteria were fractures of the skull, lethal head trauma, burn injuries, and acquired or congenital abnormalities. The study protocol was approved by the ethics committee of our university (2121264NI).

#### Western Australian population

The sample comprises MDCT scans of 225 adult individuals (112 female patients, mean age = 40.47 ± 12.99 years; 113 male patients, mean age = 37.97 ± 12.67 years) at one of the major Western Australian hospitals for clinical cranial evaluation between September 2010 and May 2011. In accordance with the National Statement on Ethical Conduct in Human Research (National Statement), the scans were anonymized, with only sex and age data retained. Although specific information on the ethnicity of each individual was not maintained in the patient data, the entire sample was taken as representative of a “‘typical” Western Australian population [19]. Individuals with obvious congenital or acquired cranial pathology were excluded if it affected their normal morphology and/or ability to accurately locate necessary cranial landmarks. Research ethics approval was granted by the human research ethics committee of our university (2020/ET000038).

### Methods

For Japanese subjects, PMCT scanning was performed with a 16-row detector CT system (Eclos; Fujifilm Healthcare Corporation, Tokyo, Japan). The scanning protocol was as follows: collimation of 0.625 mm, reconstruction interval of 0.625 mm, tube voltage of 120 kV, and tube current of 200 mA.

For Western Australian subjects, cranial imaging was performed using a 64-slice CT scanner (Brilliance; Phillips Healthcare, NSW, Australia) with an average slice thickness of 0.90 mm, tube voltage of 120–140 kV, and automatic tube current modulation (235–423 mA). The images were reconstructed to the same thickness.

**Fig. 1:**
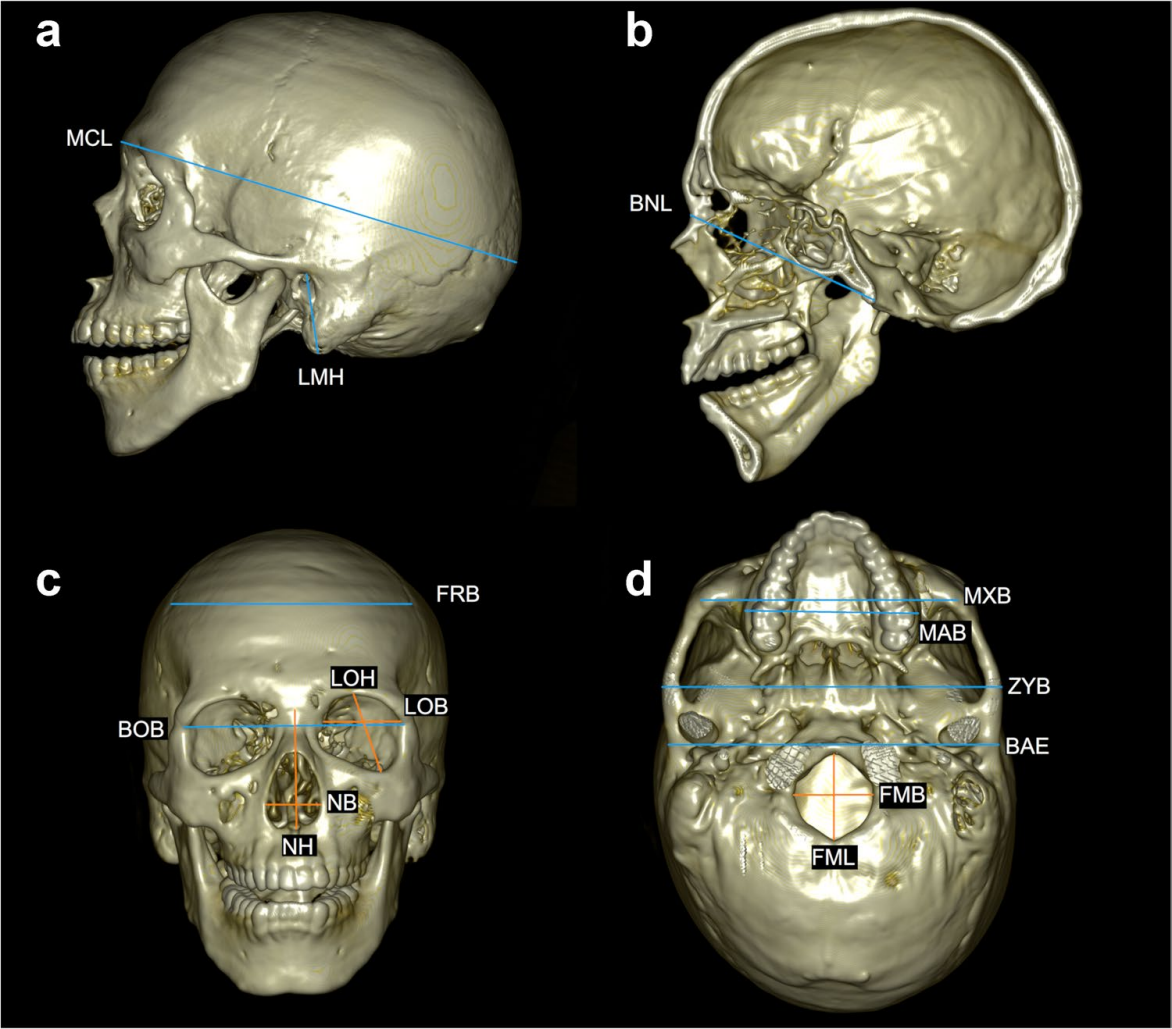
Three-dimensional computed tomography images showing cranial measurements (see Table 2 for definition): **a** maximum cranial length (MCL) and left mastoid height (LMH); **b** basion-nasion length (BNL); **c** frontal breadth (FRB), biorbital breadth (BOB), left orbit height (LOH), left orbit breadth (LOB), nasal height (NH), and nasal breadth (NB); **d** bimaxillary breadth (MXB), maxillo-alveolar breadth (MAB), bizygomatic breadth (ZYB), biauricular breadth (BAE), foramen magnum length (FML), and foramen magnum breadth (FMB). Right mastoid height (RMH), right orbit height (ROH), or right orbit breadth (ROB) is not shown because they are just left symmetrical

Image data processing and three-dimensional (3D) volume rendering were performed on a workstation (OsiriX MD version 11.0.2; Pixmeo SARL, Geneva, Switzerland). Soft tissue kernel was used for the acquisition of the CT. In accordance with previous research [19–25], 35 cranial landmarks (Table 1) were acquired on each sample. Thereafter, 18 measurements (Table 2; Fig. 1) were calculated based on coordinates of the landmarks obtained in 3D images using MorphDB (an in-house developed database application) and the Excel software (Microsoft Office 2019, Microsoft, Redmond, Washington, USA).

**Table 1:** Definitions of the landmarks

Landmark	Definition
Bilateral landmarks	
Frontoparietal temporale (fpt) [20]	Frontoparietal (coronal) suture at the intersection of the superior temporal line
Mastoidale (ms) [21]	The most inferior point on the mastoid process
Zygion (zy) [21]	The most lateral point on the zygomatic arch
Lateral foramen magnum (fml) [19]	The point of greatest lateral curvature of the foramen magnum
Porion (po) [20]	The highest point on the superior margin of the external auditory meatus
Alare (al) [20]	The most lateral point on the nasal aperture
Supraorbitale (s) [22]	The point on the orbital margin in line with the most lateral supraorbital foramen or notch
Orbitale (or) [21]	Lowest point in the margin of the orbit
Dacyron (d) [23]	The point at which the sutures between the frontal, maxillary and lacrimal bones meet
Inferior lateral zygomatic (ifz) [19]	The most inferior, lateral point on the anterior portion of the zygomatic bone
Zygofacial orbitale (zfo) [20]	Point on the orbital margin closest to the most posterior zygomatic-facial foramen
Ectomolare (ecm) [21]	The most lateral point on the buccal surface of the alveolar margin. Generally positioned on the alveolar margin of the second maxillary molar
Frontozygomatic orbitale (fo) [20]	Frontozygomatic suture at the orbital margin
Articular eminence (ae) [20]	The lateral edge of the articular eminence
Midline landmarks	
Glabella (g) [23]	The most anterior point in the mid-sagittal plane of the bony prominences joining the superciliary ridges
Opisthocranion (op) [21]	The most posterior point on the skull not on the external occipital protuberance
Basion (ba) [24]	The point at which the anterior border of the foramen magnum is intersected by the mid-sagittal plane
Nasion (n) [24]	The point of intersection of the naso-frontal suture and the mid-sagittal plane
Bregma (b) [24]	The posterior border of the frontal bone in the mid-sagittal plane, usually the junction of the coronal and sagittal sutures on the frontal bone
Opisthion (o) [21]	The midpoint of the posterior margin of the foramen magnum in the mid-sagittal plane
Inferior nasal spine (ins) [20]	Intermaxillary suture at the inferior margin of the nasal aperture at the tip of the nasal spine

**Table 2:** Definitions of the measurements

Measurement	Landmarks	Definition
Maximum cranial length (MCL) [24]	g-op	The straight-line distance from glabella to opisthocranion in the mid-sagittal plane
Basion-nasion length (BNL) [24]	ba-n	The distance between basion and nasion
Frontal breadth (FRB) [19]	fpt-fpt	Breadth at the coronal suture, perpendicular to the median plane at the temporal line
Bizygomatic breadth (ZYB) [25]	zy-zy	The maximum breadth across the zygomatic arches, perpendicular to the mid-sagittal plane
Foramen magnum length (FML) [25]	ba-o	The mid-sagittal distance from opisthion to basion
Foramen magnum breadth (FMB) [25]	fml-fml	Distance between the lateral margins of the foramen magnum at the point of greatest lateral curvature
Left mastoid height (LMH) [24]	po-ms	The direct distance between left porion and left mastoidale
Right mastoid height (RMH) [24]	po-ms	The direct distance between right porion and right mastoidale
Nasal height (NH) [21]	n-ins	Average height from nasion to the lowest point on the border of the nasal aperture on either side
Nasal breadth (NB) [24]	al-al	Distance between the anterior edges of the nasal aperture at its widest extent
Left orbit height (LOH) [24]	s-or	Height between the upper and lower borders of the left orbit
Right orbit height (ROH) [24]	s-or	Height between the upper and lower borders of the right orbit
Left orbit breadth (LOB) [19]	zfo-d	Breadth from dacryon to zygofacial approximating the longitudinal axis that bisects the left orbit into equal upper and lower parts
Right orbit breadth (ROB) [19]	zfo-d	Breadth from dacryon to zygofacial approximating the longitudinal axis that bisects the right orbit into equal upper and lower parts
Bimaxillary breadth (MXB) [24]	ifz-ifz	Breadth across the maxilla between zygomaxillare
Maxillo-alveolar breadth (MAB) [24]	ecm-ecm	The maximum breadth across the alveolar borders of the maxilla measured on the lateral surfaces at the location of ectomalare
Biorbital breadth (BOB) [19]	fo-fo	Breadth across the face between the most anterior point on the frontomalare suture on either side
Biauricular breadth (BAE) [24]	ae-ae	The least exterior breadth across the roots of the zygomatic processes

A subset of six subjects (three females and three males) was randomly selected; the original author recollected the subset data to assess intra-observer error; another co-author collected the subset data to assess inter-observer error. All 35 cranial landmarks were acquired on each of six subjects, and this process was repeated a total of six times, with a minimum of two days interval. In an effort to mitigate recall between repetitions, landmark acquisition order was varied each time. The relative technical error of measurements (rTEM, %) and coefficient of reliability (*R*) were then calculated. The acceptable rTEM range as outlined by established anthropological research [26–28] was < 5%; an *R* value > 0.75 was considered sufficiently precise [21, 29].

Descriptive statistics including mean, standard deviation, and range were calculated to provide an overview of the sample. The Kruskal-Wallis test was used to compare the measurements of the four groups (Japanese and Western Australian female and male); a *p* value of <0.05 was considered statistically significant. A series of post hoc Mann-Whitney *U* test was used for between-groups comparisons with Bonferroni correction after the Kruskal-Wallis test. Two machine learning methods (random forest modeling, RFM; support vector machine, SVM) were used to classify ancestry. RFM belongs to a class of machine learning techniques that consist of traditional classification trees created using a nonparametric algorithm that incorporates majority voting and bagging to assign cases to response classes [30–32]. Bagging is a machine learning ensemble meta-algorithm that generates multiple new training sets by sampling (replacing) the original data, reducing the variance between observations and the potential for overfitting, and improving model stability and classification accuracy [33]. The latter facilitates an estimate of out-of-bag error, which provides an unbiased estimate of the generalization ability of the random forest compared to K-fold cross-validation [34].

SVMs generate classification rules by maximizing the margin between two groups using data located at the edges of the multivariate space (the intersection of two groups). This method identifies support vectors to define a classifier that maximizes classification accuracy, and thus, small sample sizes or outlier values do not affect SVMs [35]. The number of support vectors is directly related to the predictability of the model, with a higher number of support vectors indicating less separable data [36].

The utility of machine learning models was examined in three scenarios: (i) a two-way model distinguished by ancestry (without considering sex), (ii) a four-way model distinguished by ancestry and sex simultaneously, and (iii) two-way models distinguished by sex-specific (female and male) population. The random forest feature importance was calculated during the analysis. All machine learning performances were analyzed using R 4.2.3 (R Foundation for Statistical Computing, Vienna, Austria) with the “randomForest” and “e1071” packages [37, 38].

## Results

As shown in Table 3, the rTEMs and the *R* values ranged from 0.41 to 2.66% and from 0.785 to 0.993, respectively. The mean, standard deviation, and ranges of the 18 measurements are shown in Table 4. Among Japanese individuals, all of the mean measurement values in male subjects are larger than the corresponding mean measurements for female subjects. Among the Western Australian individuals, mean male values were greater than females for all measurements, except FRB. Among the same sexes, the mean values of some measurements (e.g., MCL, BNL, and FRB) were larger in Western Australian compared to Japanese individuals. Conversely, the mean values of ZYB, LMH, RMH, and NH were slightly larger in Japanese individuals. The Kruskal-Wallis test showed significant differences in all of the measurements between the four groups (*p* < 0.001). The results of the post hoc tests comparing the measurements of each two groups are given in Online Resource 1.

**Table 3:** Relative technical error of measurements (rTEM) and coefficient of reliability (*R*)

Measurement	Intraobserver error		Interobserver error	
	rTEM	*R*	rTEM	*R*
MCL	0.41	0.987	0.68	0.965
BNL	0.63	0.979	0.63	0.980
FRB	0.92	0.993	1.84	0.785
ZYB	0.57	0.952	0.61	0.974
FML	1.11	0.978	1.34	0.890
FMB	1.01	0.912	1.35	0.839
LMH	2.59	0.931	2.66	0.830
RMH	0.72	0.971	1.20	0.880
NH	0.99	0.895	1.42	0.851
NB	1.25	0.982	2.13	0.968
LOH	0.98	0.944	1.60	0.794
ROH	0.72	0.981	1.76	0.878
LOB	1.91	0.925	2.33	0.855
ROB	1.87	0.932	2.14	0.849
MXB	1.65	0.925	1.71	0.886
MAB	1.28	0.960	1.54	0.937
BOB	1.34	0.982	0.65	0.980
BAE	0.62	0.948	1.46	0.904

**Table 4:** Descriptive statistics of 18 cranial measurements

Measurement	Japanese				Western Australian			
	Female (*n* = 111)		Male (*n* = 119)		Female (*n* = 112)		Male (*n* = 113)	
	Range	Mean ± SD^a^	Range	Mean ± SD	Range	Mean ± SD	Range	Mean ± SD
MCL (mm)	158.74–192.72	171.83 ± 5.89	170.33–201.04	183.78 ± 6.10	164.50–195.24	179.46 ± 6.31	170.08–205.07	189.99 ± 7.07
BNL (mm)	89.91–107.38	99.62 ± 3.54	94.59–124.42	106.38 ± 4.16	90.06–109.04	100.42 ± 3.96	95.51–117.97	107.33 ± 4.64
FRB (mm)	85.36–119.27	104.09 ± 6.19	93.87–127.14	106.62 ± 6.09	91.82–126.78	111.11 ± 7.12	90.07–127.65	110.00 ± 7.36
ZYB (mm)	122.98–138.97	130.92 ± 3.91	124.88–150.76	139.77 ± 4.91	114.27–135.22	123.22 ± 3.97	121.92–138.43	131.32 ± 4.01
FML (mm)	29.73–40.35	34.77 ± 2.21	29.22–41.97	36.60 ± 2.24	32.09–43.26	36.59 ± 2.27	30.53–43.21	37.71 ± 2.31
FMB (mm)	24.03–33.56	28.92 ± 1.90	25.19–36.10	30.47 ± 1.79	24.81–37.04	31.09 ± 2.20	26.39–37.56	31.78 ± 2.14
LMH (mm)	21.17–36.06	29.58 ± 3.07	27.29–41.21	34.10 ± 2.91	19.42–36.72	28.70 ± 3.65	25.08–41.47	33.00 ± 3.42
RMH (mm)	22.07–36.92	29.44 ± 3.25	28.25–41.54	34.49 ± 2.90	20.31–38.76	29.24 ± 3.68	24.67–43.01	33.24 ± 3.66
NH (mm)	42.67–62.33	52.57 ± 3.45	48.07–62.51	55.71 ± 2.81	43.93–58.38	50.30 ± 2.93	45.22–60.12	53.51 ± 3.11
NB (mm)	20.81–32.76	25.92 ± 2.17	22.20–31.41	26.89 ± 1.94	19.13–29.26	23.57 ± 2.18	20.06–31.08	24.53 ± 2.03
NOH (mm)	36.63–45.71	41.06 ± 1.90	37.47–47.50	41.98 ± 1.91	34.31–45.94	40.53 ± 2.10	36.01–48.13	42.15 ± 2.38
ROH (mm)	36.78–48.25	40.62 ± 2.13	34.72–46.21	41.04 ± 2.14	34.27–44.81	39.92 ± 2.14	35.34–47.70	41.32 ± 2.64
LOB (mm)	34.07–43.27	37.93 ± 1.63	34.99–44.12	39.39 ± 1.60	35.30–44.66	38.78 ± 1.62	35.51–44.64	39.50 ± 1.89
ROB (mm)	34.72–42.33	37.74 ± 1.47	35.74–43.95	39.31 ± 1.47	35.65–44.94	38.71 ± 1.60	35.44–44.84	39.38 ± 1.89
MXB (mm)	83.42–103.10	95.11 ± 3.90	84.70–108.43	98.72 ± 5.18	73.71–94.40	84.11 ± 4.70	79.46–105.34	91.93 ± 4.89
MAB (mm)	50.52–70.73	61.09 ± 3.62	52.90–75.37	65.72 ± 4.11	48.85–71.42	58.16 ± 4.34	50.24–73.00	61.76 ± 4.33
BOB (mm)	88.12–108.08	96.03 ± 3.11	91.79–109.59	100.79 ± 3.65	85.00–107.22	94.28 ± 3.85	92.37–109.84	99.30 ± 3.62
BAE (mm)	109.98–132.19	120.12 ± 4.37	110.20–137.55	127.43 ± 4.94	104.07–129.46	115.02 ± 4.22	108.73–132.82	120.80 ± 4.15

^a^Standard deviation

Results of machine learning models are summarized in Tables 5–8. As shown in Table 5, the accuracy of the two-way unisex model was 93.2% for RFM and 97.1% for SVM, respectively. Accuracy was higher in the Japanese, compared to the Western Australian sample. The four-way model demonstrated an overall classification accuracy of 84.0% for RFM and 93.0% for SVM (Table 6). Female individuals were more likely to be correctly classified according to sex. The sex-specific ancestry analyses also revealed that the correct classification rates were higher in the female (95.1% for RFM and 100% for SVM) than in the male samples (91.4% for RFM and 97.4% for SVM; Tables 7 and 8).

**Table 5:** Classification matrix showing classification of groups according to ancestry

Group	RFM			SVM		
	JP	WA	% Correct	JP	WA	% Correct
JP	218	12	94.8	227	3	98.7
WA	19	206	91.6	10	215	95.6
All			93.2			97.1

*RFM* random forest modeling, *SVM* support vector machine, *JP* Japanese, *WA* Western Australian

**Table 6:** Classification matrix showing classification of groups according to ancestry and sex

Group	RFM					SVM				
	JPF	JPM	WAF	WAM	% Correct	JPF	JPM	WAF	WAM	% Correct
JPF	95	9	4	3	85.6	105	4	0	2	94.6
JPM	8	100	0	11	84.0	7	111	0	1	93.3
WAF	5	0	95	12	84.8	0	0	106	6	94.6
WAM	2	8	11	92	81.4	1	6	5	101	89.4
All					84.0					93.0

*JPF* Japanese female, *JPM* Japanese male, *WAF* Western Australian female, *WAM* Western Australian male

**Table 7:** Classification matrix showing classification of groups according to sex-specific ancestry (female)

Group	RFM			SVM		
	JPF	WAF	% Correct	JPF	WAF	% Correct
JPF	108	3	97.3	111	0	100
WAF	8	104	92.9	0	112	100
All			95.1			100

**Table 8:** Classification matrix showing classification of groups according to sex-specific ancestry (male)

	RFM			SVM		
Group	JPM	WAM	% Correct	JPM	WAM	% Correct
JPM	108	11	90.8	118	1	99.2
WAM	9	104	92.0	5	108	95.6
All			91.4			97.4

**Fig. 2:**
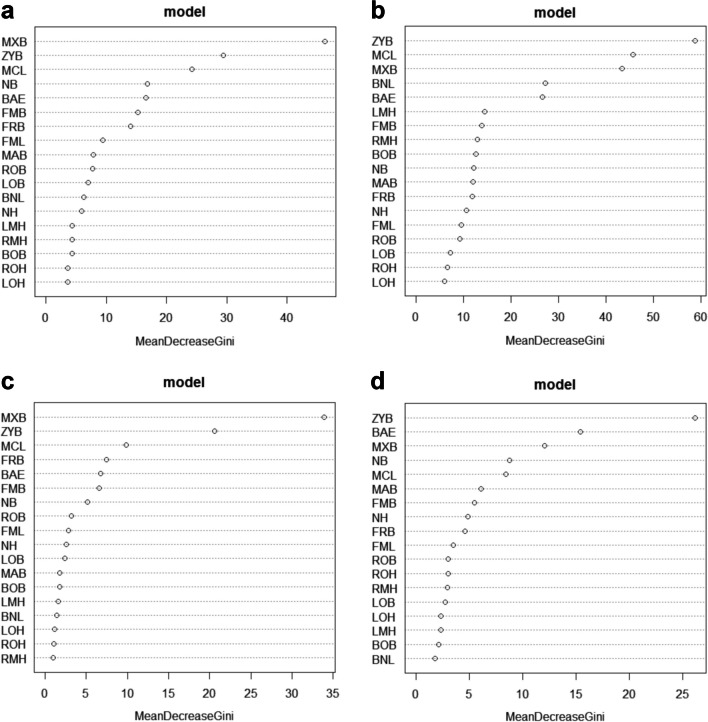
Random forest feature importance (mean decrease Gini) for the response variable. **a** The two-way unisex model, **b** the four-way sex and ancestry model, **c** the two-way female model, and **d** the two-way male model

Random forest feature importance demonstrated that MCL, ZYB, MXB, and BAE ranked in the top five in all analyses, indicating that they are the strongest weighted measurements (express the greatest population variance) relative to achieving correct classifications (Fig. 2; Online Resource 2).

## Discussion

In the present study, the intra- and inter-observer errors were small and likely to be negligible. Considering these results, cranial landmark acquisition using 3D CT images in this study is highly reproducible.

Cranial size and shape are known to express significant populational variability [39–41]. Previous research has reported that the skulls of Australian individuals are on average longer, taller, and with narrower frontal bones than those of Japanese individuals [19, 42, 43]. The results of this study also showed that the mean values of MCL and BNL were larger in Western Australian subjects, whereas the mean values of LMH and RMH were larger in Japanese subjects. However, the mean values of FRB were larger for Western Australian individuals, which did not accord with previous findings.

The results of this study revealed that the correct classification rates of the Japanese and Western Australian individuals were greater than 90% when sex was not considered, and above 80% when sex was classified simultaneously. This clearly indicates that cranial measurements derived from CT images are useful for the classification of Japanese and Western Australian individuals. Franklin and Flavel [44] reported that Australia has become a multicultural country, with a dynamic population demographic that includes considerable migration from southeast Asia, with intra-population variation also evident between the States and Territories. Irrespective, the results of this study suggest that Japanese and Western Australian populations have different skull shapes.

In the present study, the mean age of the Japanese individuals was higher than that of the Western Australian subjects. Previous research has noted an increase in the size of some cranial regions in middle-aged to elderly individuals; it has accordingly been suggested that large differences between age distributions may skew results [45]. Conversely, Albert et al. [46] reported modest increases in craniofacial dimensions (1.1–1.6 mm) in the elderly, with facial height presenting the largest change relative to antemortem tooth loss. Therefore, although the effects of age-related craniofacial remodeling should be recognized, age may not be expected to be a major contributor to the misclassification rate observed in this study.

Hefner et al. [11] achieved 89.6% accuracy based on applying RFM to 110 skulls representing modern American White (*n* = 72), African American (*n* = 38), and Southwestern Hispanic (*n* = 39) skulls; the important craniometric variables in the RFM included MCL and PBL. Navega et al. [10] used AncesTrees, which is a statistical procedure using RFM comprising 23 craniometric variables from 1734 individuals, representative of six major ancestral groups (European, African, Austro-Melanesian, Polynesian, Native American, and East Asian). The program was tested in 128 adult crania (32 individuals of African ancestry and 96 of European ancestry); 75% of the African and 79.2% of the European individuals were correctly identified. The model involving only African and European ancestral groups was more accurate (93.8%). Navega et al. [10] also reported that ZYB and BAE are the important variables in the RFM for ancestry and sex estimation. Similarly, our study demonstrated that MCL, ZYB, and BAE were the important factors (Fig. 2). Furthermore, there were significant differences in these variables between each two groups except for ZYB and BAE between Japanese female and Western Australian male groups, indicating that these measurements are useful in the classification of ancestry in multiple global populations.

Hefner and Ousley [47] also reported that RFM demonstrated an overall classification rate of 85.5% for ancestry in a sample of 543 Americans (African American, Hispanic and White). The most significant advantage of RFM is that it transforms a low-bias and high-variance model into a low-bias and low-variance model by training multiple decision trees simultaneously; the low variance is the most valuable feature for anthropological application [10]. Although LDA is also a valuable method to perform ancestry estimation from metrical data, it can usually be outperformed by the latest machine learning classification algorithms [11, 48–50].

Spiros and Hefner [35] and Hefner and Ousley [47] reported that the SVM model provided higher classification accuracy than the RFM for the American individuals. Nikita and Nikitas [51] also reported that the SVM is more effective than RFM for skeletal ancestry and sex assessment. In this study, SVM revealed higher correct classification rates than RFM, probably due to the relatively small amount of data. Further studies considering other machine learning methods are necessary in the future.

In this study, when only female samples were considered, the correct classification rates according to ancestry were over 95%. Therefore, it is hypothesized that if an unidentified skull can be presumed to be female, it may be possible to estimate ancestry more accurately. However, other studies on sex-specific ancestry estimation using the skull are scarce and further research is required.

The majority of previous craniometric research specific to the estimation of ancestry have involved the analysis of data acquired in physical specimens [10, 52]. The data in the present study are, to the best of our knowledge, amongst the first to assess the feasibility of ancestry estimation using 3D CT images of the skull. Non-invasive imaging techniques can maintain and visualize the arrangement of spatial structures and their potential relationships [53]. Previous research has considered the reliability and accuracy of estimating other biological attributes, such as sex, age, and stature in CT images [19, 54–57]. Sharing CT data among facilities in various countries should facilitate collection of global and contemporary multi-populational data and thus afford a deeper understanding of craniometric diversity relative to ancestral origin.

Regarding skeletal measurements for ancestry estimation, it should be recognized that some populations are poorly described in the published literature. Therefore, more comprehensive databases of missing persons are required to enhance identification efforts. In addition, it is crucial to consider that cranial features and measurements are phenotypic characteristics that are partially determined by heritability and influenced by the environment [58], and as noted above, are changing through time and especially with increased admixture in contemporary populations.

The literature clearly indicates that the majority of forensic anthropology ancestry studies focused broadly on the skull, despite bones such as the femur and tibia also potentially providing useful information [3]. Thus, further research addressing other skeletal measurements based on CT imaging is needed to assess the feasibility of ancestry estimation.

This study demonstrated several limitations. First, data were collected from two different facilities using 16- and 64-row detector CT systems, with different conditions for the reconstructed images. Although these issues were not expected to significantly affect the measurements, it would be more appropriate to use the same detector CT images under the same conditions. Second, PMCT data and CT data from living patients were used in this study. Although it is unlikely that the shape or measurements change significantly between ante- and post-mortem human remains, the difference was not investigated in the present study. Third, morphometric geometric analysis may detect other significant differences by detailing differences due to cranial size and shape [59, 60].

## Conclusions

This study demonstrated that cranial measurements derived in 3D CT images are useful for the accurate statistical classification of Japanese and Western Australian individuals. This is the first study to investigate the feasibility of ancestry estimation using 3D CT images of cranial measurements. Further CT data involving other populations should be collected to enable research of more diverse populations across the globe. In addition, further research addressing other skeletal measurements based on CT imaging to estimate ancestry is required.

### Supplementary information

ESM 1

ESM 2
